# Quantitative evaluation of digital-image enhancement during heads-up surgery

**DOI:** 10.1038/s41598-019-52492-z

**Published:** 2019-11-04

**Authors:** Kunihiko Akiyama, Ken Watanabe, Masaki Fukui, Hiroshi Higuchi, Toru Noda

**Affiliations:** 1 Department of Ophthalmology, National Hospital Organization Tokyo Medical Center, 2-5-1, Higashigaoka, Meguro-ku, Tokyo, 152-8902 Japan; 2 Division of Vision Research, National Institute of Sensory Organs, National Hospital Organization Tokyo Medical Center, 2-5-1, Higashigaoka, Meguro-ku, Tokyo, 152-8902 Japan; 3 Sony Corporation, 1-7-1, Konan, Minato-ku, Tokyo, 108-0075 Japan

**Keywords:** Translational research, Imaging techniques

## Abstract

Image-processing is an advantage of heads-up surgery and expected to facilitate ophthalmic surgeries. To evaluate image-processing quantitatively, we analyzed the surgical images of twenty eyes that underwent vitrectomy with internal limiting membrane (ILM) peeling assisted by Brilliant Blue G (BBG). Still images of the peeling procedure were obtained from the surgical video, and the color difference was calculated between two adjacent spots inside and outside the ILM-peeling contour, i.e., without and with BBG staining, respectively. The color differences were compared between the two settings with and without image-processing, delivered by an algorithm to enhance the color and contrast. Color differences were calculated using two methods: the Euclidean distance based on RGB values (RGB distance) and the Delta-E00 formula provided by the International Commission on Illumination. In five cases, minimum light intensities required to recognize the contour of ILM-peeling were compared during surgeries between the two settings with and without enhancement. Image-processing increased the mean color difference significantly (*P* < 0.001) from 15.47 and 4.49 to 34.03 and 8.00, respectively, for the RGB distance and Delta-E00. The minimum light intensity was reduced from 15 to 5 on average by image-enhancement. These results showed image-processing enhances color differences and reduces light intensities during vitrectomy.

## Introduction

Digital three-dimensional (3-D) displays have been introduced recently for use during ophthalmic surgeries, during which surgeons observe surgical views on a display instead of through the eye pieces of a surgical microscope^1–9^. This evolutionary technique often is referred to as heads-up surgery (HUS) based on the posture of the operating surgeon. The technical advantage of HUS is digital enhancement of the surgical images, which potentially facilitates surgical procedures if presented with minimal delay after the real-time surgical view.

Image enhancement for HUS usually considers brightness, contrast, and color using software intrinsic to the system^1^. Although some surgeons have claimed the advantages of this technology^1–3,7,8^, those effects have not been fully analyzed quantitatively. In the current study, we assessed the enhancement effect on visualization of membrane staining during vitrectomy using the software built into a display commercially available for medical use, and the effect of the enhancement to reduce the light intensity required for visualization of the surgical view.

## Material and Methods

### Study design

This study was a retrospective case series that evaluated surgical videos of patients who underwent vitrectomy with internal limiting membrane (ILM) peeling from April 1 to August 31, 2017, at the National Hospital Organization Tokyo Medical Center. The minimum light intensity for observation of ILM was also evaluated during surgery in some cases. All procedures performed in this study were in accordance with the 1964 Helsinki declaration and its later amendments or comparable ethical standards. Informed consent was obtained from all individual participants included in the study. The Institutional Review Board of the National Hospital Organization Tokyo Medical Center approved the study protocol (approval number, R17-050).

The inclusion criteria required that the surgeries were performed on the HUS platform, that ILM peeling was performed successfully after staining with Brilliant Blue G (BBG)^10^, and that surgical images of the ILM peeling with appropriate focusing and image quality were available for assessment. The exclusion criteria included severe atrophy or scarring of the macula that might prevent appropriate visualization of the ILM peeling and corneal opacity or severe cataract that might have negatively affected the recorded surgical images.

### Patients and observations

The surgical videos of the vitrectomies performed in 20 eyes of 20 patients (mean age, 64.4 ± 8.1 years) were collected. Validity of the number of the included cases was confirmed using the statistical power analysis based on the parameters shown in results and Table 1 (required sample size: 10, 11, 18, 9 and 10 for R, G, B, RGB distance and delta E00, respectively, to reach the statistical power 0.8). The surgeries were performed to treat epiretinal membranes (ERMs) (11 cases); foveal schisis (3 cases); and macular holes, rhegmatogenous retinal detachments (RRDs), and optic pit maculopathy (2 cases each). In all cases, the ILMs were peeled smoothly. RRDs were macula-sparing in both cases, and there was no abnormal finding such as an ERM in the macula; ILM was peeled to prevent post-surgical ERM growth^11^. One case with RRD was pseudo phakic. The other cases were phakic and were treated by the triple procedure combined with cataract surgery. Consequently, the lens status during ILM peeling procedure was pseudo phakic in one case and aphakic in 19 cases, as IOLs were implanted at the end of the surgery in these cases. Endo-illumination was provided by a xenon light source (Constellation Illuminator, Alcon Laboratories, Hünenberg, Switzerland) with a handheld light (Straight Endoilluminator 25 + , Alcon Laboratories, Fort Worth, TX) during the ILM peeling procedure.

**Table 1:** Comparison of RGB values, RGB distance, and delta-E00 between enhancement on and off.

Methods	A.I.M.E.-off^a^	A.I.M.E.-on^a^	Difference between A.I.M.E.-on and -off	95% CI of the difference	*P* value^b^
R	10.10 ± 5.60 (80.65 ± 24.50/ 90.75 ± 25.18)	22.95 ± 10.80 (157.15 ± 42.34/ 180.10 ± 45.32)	12.85 ± 7.10	9.53–16.17	< 0.001
G	8.15 ± 4.18 (79.70 ± 27.40/ 87.85 ± 28.07)	18.50 ± 9.74 (154.95 ± 43.15/ 173.45 ± 45.70)	10.35 ± 6.75	7.19–13.51	< 0.001
B	6.85 ± 4.55 (97.40 ± 36.61/ 103.15 ± 35.72)	14.50 ± 9.92 (164.85 ± 42.70/ 177.15 ± 42.15)	7.65 ± 6.60	4.56–10.74	< 0.001
RGB distance	15.47 ± 6.71	34.03 ± 15.06	18.59 ± 10.79	13.53–23.63	< 0.001
Delta E00	4.49 ± 2.21	8.00 ± 2.68	3.51 ± 1.21	2.94–4.08	< 0.001

A.I.M.E. = Advanced Image Multiple Enhancer; CI = confidence interval.

^a^Color difference (values with/without Brilliant Blue G staining) for R, G and B.

^b^Two-tailed paired *t*-test.

Each value is expressed as mean ± standard deviation.

All surgeries were performed as HUS procedures and viewed on a 3-D 4 K monitor for medical use (LMD-X550MT, Sony Corporation, Tokyo, Japan), which was connected to the surgical microscope (OMS-800, Topcon Corporation, Tokyo, Japan) via two CCD cameras (MKC-300HD, Ikegami Tsushinki Co., Ltd., Tokyo, Japan) attached to the beam splitter built-in the microscope. The splitting ratio was 20% to the cameras and 80% to the eye pieces. The auto-iris function was switched on, and the automatic gain control mode was off during the surgeries. An intrinsic image-processing algorithm, i.e., Advanced Image Multiple Enhancer (A.I.M.E., Sony Corporation), was activated intraoperatively for simultaneous image enhancement. The two-dimensional surgical view was recorded via a camera on the right side, because the monitor was not equipped with a recording device. Therefore, the recorded images were the original data without image processing by the A.I.M.E. The quality of images observed by each eye was considered equivalent to each other, because the optical properties of the barrels on both sides of the microscope were equal and the images from both sides were enhanced equally by A.I.M.E. algorithm, as certified by the manufacturer.

**Figure 1:**
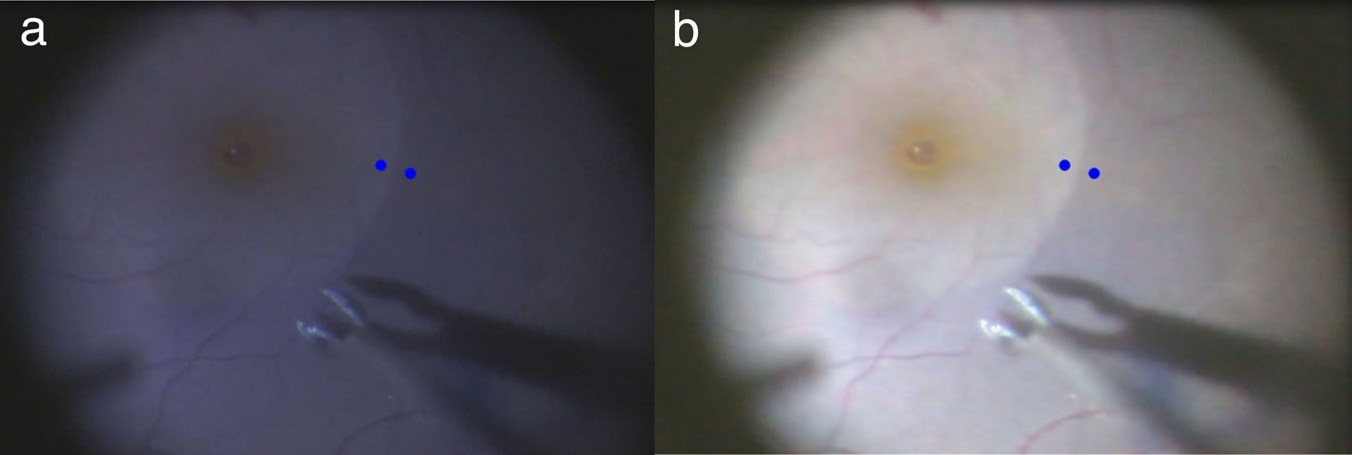
Images of internal limiting membrane (ILM) peeling facilitated by Brilliant Blue G (BBG) staining. A still image without image processing is obtained from the surgical video (**a**) and enhanced for color and contrast using the image-processing software built into the three-dimensional display used for heads-up surgery (**b**). The blue points indicate the two adjacent spots inside and outside of the ILM peeling contour, i.e., without and with BBG staining, respectively. These two spots are at exactly the same coordinates in both images. The color differences caused by BBG staining are calculated between the two spots and compared between images before (**a**) and after enhancement (**b**).

For analyses, a still image of the macula during ILM peeling was obtained as a snapshot from the recorded video (A.I.M.E.-off) (Fig. 1a). To assess the enhancement effect, the obtained image was processed by the A.I.M.E. algorithm for color and contrast (A.I.M.E.-on) (Fig. 1b). On each image, two adjacent spots were selected that were inside and outside of the contour of the circular area of ILM peeling, i.e., without and with BBG staining, respectively (Fig. 1a,b), and the color difference between these spots was calculated by 8-bit conversion. Those two spots were fixed at the same coordinates on both the paired A.I.M.E.-on and A.I.M.E.-off images in each case.

### Assessment of the color differences

Two methods were used to evaluate the color differences, i.e., the Euclidean distance of the two spots in the color space identified with the RGB values (RGB distance) and the Delta-E00 formula^12,13^. The RGB distance was defined as:

$${\rm{RGB}}\,{\rm{distance}}\,=\sqrt{{({R}_{2}-{R}_{1})}^{2}+{({G}_{2}-{G}_{1})}^{2}+{({B}_{2}-{B}_{1})}^{2}}$$

where *RGB*_*1*_ *and*_*2*_ represent the values of each of the two spots to be compared.

The International Commission of Illumination provided and recommended the Delta-E00 formula to assess the color differences most recently; the formula was reported to be more effective than other previous formulas for detecting large, small, or threshold color differences^14^. This formula is based on the CIELAB color space, which is defined by luminosity and color opponents (green-red and blue-yellow), and includes correction of the Euclidean distance between two color points based on the characteristics of human perception of luminosity, hue, chroma, and interactive terms between the hue and chroma differences^12,13^. The Delta-E00 formula is expressed as follows, with several equations to calculate each factor that appears in the formula (not presented here; see references for details)^12,13^.

$${\rm{Delta}}-{\rm{E}}00\,=\,\sqrt{{(\frac{\varDelta L^{\prime} }{{k}_{L}{S}_{L}})}^{2}+{(\frac{\varDelta C^{\prime} }{{k}_{C}{S}_{C}})}^{2}+{(\frac{\varDelta H^{\prime} }{{k}_{H}{S}_{H}})}^{2}+{R}_{T}(\frac{\varDelta C^{\prime} }{{k}_{C}{S}_{C}})(\frac{\varDelta H^{\prime} }{{k}_{H}{S}_{H}})}$$

*k*_*L*_, *k*_*C*_ and *k*_*H*_: parametric weighting factors*L*, *C* and *H*: luminosity, chroma and hue, respectively

The RGB values and coordinates of the analyzed spots were specified on the viewer software, and the values were translated into the luminosity and color opponents for the serial calculations of Delta-E00, using parameters based on BT.709 for high-definition television (defined by the International Telecommunication Union-Radiocommunication Sector)^15^, after inverse gamma correction (gamma = 2.2) and the definition of the white point values by the standard light source D65.

These two methods were applied independently to calculations of the color difference between two spots with and without BBG staining, and the color differences were compared between the A.I.M.E.-on and A.I.M.E.-off images using the two-tailed *t*-test. The effects of the underlying diseases on the color differences also were analyzed regarding either of the two methods using analysis of variance. Those analyses were performed using IBM-SPSS Statistics, Version 24.0 (IBM Corp., Armonk, NY). *P* < 0.05 was considered statistically significant.

### Minimum light intensity to distinguish BBG-stained ILM

Because the brightness of the surgical view was significantly amplified by A.I.M.E. enhancement, we conducted another observation to identify the minimum light intensity required to recognize the contour of the ILM-peeled area, and compared the results between A.I.M.E.-on and -off conditions in five cases treated for ERM.

After the ILM-peeling using BBG staining, the light intensities were changed by 5-scale step under A.I.M.E.-on and -off conditions. The surgeon and the assistant independently judged the minimum light intensity at which the contour of ILM-peeled area was distinguished, and the values with agreement by the two observers were compared between A.I.M.E.-on and -off conditions.

## Results

All patients underwent surgeries performed on the 3-D monitor with the A.I.M.E. algorithm switched on. The manufacturer reported that the delay caused by the image processing was shorter than 30 milliseconds (a reference value provided by the manufacturer; no published data available), and, therefore, the surgeons did not notice any delay during the surgical procedures. Since the A.I.M.E.-on image (Fig. 1b) was extremely bright compared to the original A.I.M.E.-off image (Fig. 1a), the light intensity was set at 30% to 35% in all cases, which was 10% to 15% lower than our usual setting for conventional eye-piece observation, although only 20% of the total light beams were delivered to the display by the beam splitter in our system.

**Figure 2:**
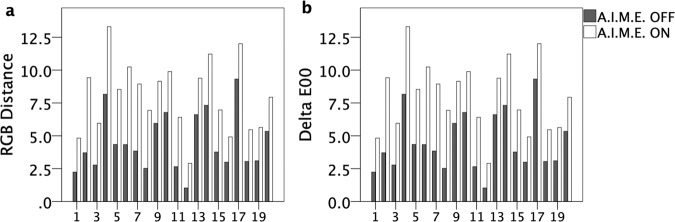
The color differences in 20 cases. The color difference is calculated by the Euclidean distance based on the RGB values (RGB distance) (**a**) and the Delta-E00 formula (**b**), the latter of which was provided and recommended by the International Commission on Illumination. The color difference is greater in all cases when enhancement with Advanced Image Multiple Enhancer algorithm (A.I.M.E.) is activated (A.I.M.E.-on).

Figure 2a,b (RGB distance and Delta-E00, respectively) showed that the color differences identified by both calculation methods clearly increased with enhancement in all cases.

**Figure 3:**
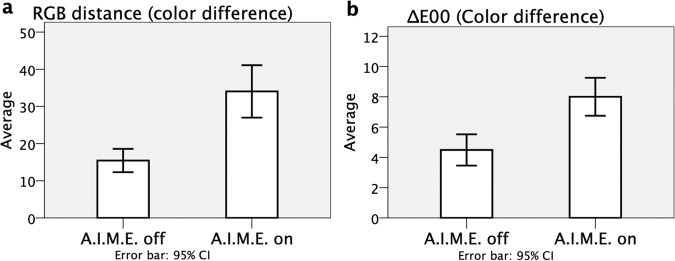
The comparison of the color differences between images before and after enhancement. The calculations are performed using two different methods as in Fig. 2: the RGB distance (**a**) and Delta-E00 formula (**b**) and expressed as the mean value and 95% confident interval. The image-processing software, the Advanced Image Multiple Enhancer (A.I.M.E.) provides the enhancement. Significant differences (*P* < 0.001, two-tailed paired *t*-test) are seen between images before (A.I.M.E.-off) and after (A.I.M.E.-on) enhancement using both calculation methods.

Table 1 shows the statistical results of the RGB values, color differences based on each method, and comparisons between the A.I.M.E.-on and A.I.M.E.-off images. The mean differences for each of the RGB values between the spots with and without BBG staining were, respectively, 10.10, 8.15, and 6.85 for the A.I.M.E.-off images and 22.95, 18.50, and 14.50 for the A.I.M.E.-on images (R, G, and B, respectively). The mean RGB distance and Delta-E00 values between the two spots were, respectively, 15.47 and 4.49 for the A.I.M.E.-off images and 34.03 and 8.00 for the A.I.M.E.-on images. A significant increase was proved in the color differences by either of the methods in the A.I.M.E.-on images (*P* < 0.001 for all comparisons). Figure 3a,b, respectively, also show the comparisons of the color differences between the A.I.M.E.-on and A.I.M.E.-off images for the RGB distance and Delta-E00.

The underlying diseases did not significantly affect the color differences calculated by any of the RGB values, RGB distance, or Delta-E00 in the A.I.M.E.-on and A.I.M.E.-off images (Table 2).

**Table 2:** Effect of underlying diseases on color differences.

Disease	No. cases	Color difference^a^				
		R	G	B	RGB distance	Delta-E00
Epiretinal membrane	11	10.18 ± 5.40/ 23.00 ± 11.82	8.36 ± 4.84/ 18.73 ± 11.85	6.18 ± 4.49/ 13.55 ± 9.72	14.98 ± 7.68/ 33.30 ± 18.01	4.42 ± 1.89/ 7.95 ± 2.36
Macular hole	2	9.50 ± 6.36/ 24.50 ± 21.92	8.50 ± 2.12/ 23.00 ± 11.31	9.50 ± 2.12/ 14.00 ± 7.07	16.47 ± 3.54/ 36.86 ± 24.31	3.66 ± 0.95/ 7.58 ± 3.76
Foveal schisis	3	15.33 ± 6.43/ 27.00 ± 8.19	10.33 ± 4.51/ 19.00 ± 8.19	5.33 ± 6.11/ 11.00 ± 12.29	20.61 ± 4.14/ 37.37 ± 2.58	6.78 ± 2.84/ 10.88 ± 1.33
Rhegmatogenous retinal detachment (macula-on)	2	5.00 ± 5.66/ 14.50 ± 2.12	5.50 ± 3.54/ 12.50 ± 2.12	5.00 ± 2.83/ 12.50 ± 0.71	9.14 ± 6.77/22.92 ± 2.11	2.06 ± 1.46/ 4.27 ± 1.92
Optic-pit maculopathy	2	7.50 ± 0.71/ 23.50 ± 0.71	6.00 ± 1.41/ 18.00 ± 4.24	12.00 ± 4.23/ 27.50 ± 13.44	15.55 ± 3.07/ 41.35 ± 6.69	4.71 ± 2.92/ 8.15 ± 2.47
*P* value^b^		0.334/0.828	0.744/0.903	0.415/0.439	0.494/0.810	0.198/0.095

A.I.M.E. = Advanced Image Multiple Enhancer.

^a^Described as A.I.M.E.-off/-on.

^b^Analysis of variance.

Each value is expressed as mean ± standard deviation.

**Figure 4:**
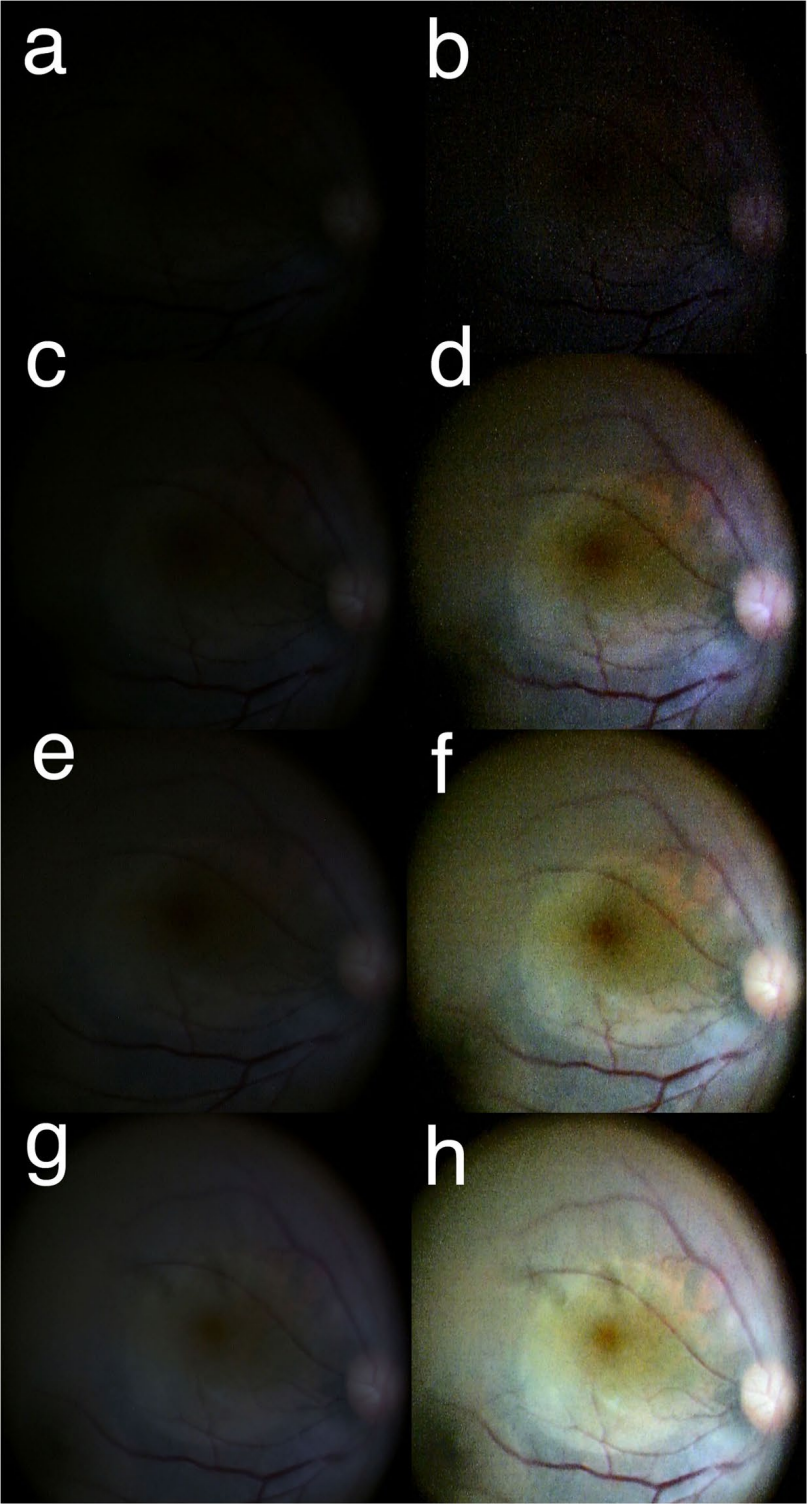
The minimum light intensity to identify the contour of internal limiting membrane (ILM) peeling using Brilliant Blue G (BBG). The original images without enhancement are shown on the left (**a,c,e,g**) and the images processed with the Advanced Image Multiple Enhancer (A.I.M.E.) are on the right side (**b,d,f,h**). The light intensity was set at 5 (**a,b**), 10 (**c,d**), 15 (**e,f**) and 20 (**g,h**) from top to bottom. The coupled images with A.I.M.E. -on and -off under each light intensity are snapshots at the same time point. With the light intensity 10, the contour is clearly recognized only when A.I.M.E. is on, while the light intensity 20 is required for visualization of the contour under A.I.M.E. -off setting. The tip of the light was fixed at a position close to the trocar and adjusted toward a proper direction to illuminate the peeled area; therefore, the distance between the light and the macula was longer than in the standard situation for ILM peeling.

The minimum light intensity for discrimination of the BBG-stained ILM is listed in Table 3. A.I.M.E. enhancement reduced the requirement of the light intensity to approximately half of that in A.I.M.E.-off condition. Figure 4 shows images of ILM peeling in Case 2 of Table 3 observed under the light intensity varying from 5 to 20. When A.I.M.E. was on (right), border of ILM-peeled area was observed clearly with the light intensity at 10, while light intensity 20 was necessary to distinguish the contour when A.I.M.E. was off (left).

**Table 3:** Minimum light intensity to distinguish BBG-stained internal limiting membrane.

Case	A.I.M.E. off	A.I.M.E. on	*p*-value^a^
1	35	15	
2	20	10	
3	25	10	
4	20	5	
5	25	10	
Ave.^b^	25.0 ± 6.1	10.0 ± 3.5	0.001

A.I.M.E. = Advanced Image Multiple Enhancer; ERM = epiretinal membrane; ILM = internal limiting membrane.

^a^Paired *t*-test.

^b^Expressed as mean ± standard deviation.

## Discussion

The effects of digital image enhancement for improving 3-D optical qualities have been reported based on the subjective impressions of the surgeons regarding resolution, color, stereopsis, illumination, and magnification^8,16^. A quantitative comparison between the digitally enhanced 3-D images and traditional microscopic observations also was performed, and the brightness of the surgical images increased as a result of digital image-processing during HUS^1^. In that report, Eckardt and Paulo described the benefits of electronic amplification for brightening the images and recognition of retinal structures that could not be seen previously^1^. Other investigators also have reported significant reductions in the minimal light intensity required for surgical maneuvers during vitrectomy using the HUS platform^2,3,7,8^.

The enhancement effect on color and contrast is another advantage of digital image processing^1^ and should contribute to better visualization of the surgical view and lower light intensity; however, objective and quantitative evaluations have not been performed previously. The current study was designed to analyze the effects of digital enhancement, which was delivered automatically and simultaneously by an image-processing algorithm built into the commercially available 3-D display for medical use. We analyzed the color differences between two spots on the retina with and without ILM staining with BBG, because ILM peeling is one of the finest maneuvers in ophthalmic surgeries and appropriate visualization of the ILM is critical to evaluate vitrectomy performed on the HUS platform. Consequently, the color differences caused by BBG staining of the ILM increased significantly with the A.I.M.E. algorithm switched on. These results were consistent with the observation by surgeons that has been revealed in previous studies, and could be explained by the functional property of the image processing algorithm. It is noteworthy that use of the A.I.M.E. algorithm also resulted in a substantial improvement in brightness, as shown by each of the RGB values with/without BBG staining, that was almost doubled when the A.I.M.E. was on (Table 1). This effect was also proved by significant reduction of the minimum light intensity required to recognize the BBG-stained ILM.

Image processing using the A.I.M.E. algorithm is based on two factors defined as color and structure, with the settings varying among eight and four steps, respectively. Because the enhanced images observed intraoperatively could not be recorded directly on the monitor, we prepared the still images obtained from the recorded video on a computer using the A.I.M.E. algorithm under the setting Color 5, Structure 1, which was similar to the actual setting during our surgeries. The structural enhancement was not raised to 2 or more to avoid increased noise and decreased resolution. These two features are subject to image enhancement and difficult to eliminate totally^1^; however, in our observation, neither was considered to cause an annoying reduction of image quality during surgery with our setting. In this respect, the adjustment scales of color and structure in the current system would be helpful for each surgeon to customize the image processing to the most comfortable level for various surgical procedures.

To assess the color differences, we used two distinct methods. The balance of the RGB color is calculated simply based on the RGB values measured physically on the viewer software, whereas those values do not correctly reflect the human visual perception, which is modified in a complicated manner with tremendous influences of brightness, hue, and chroma^12,13^. The Delta-E00 formula currently is considered the most reliable metric of color differences that suit human perception^14^. Therefore, the current results indicate the superiority of image enhancement with either of the two methods, including (Delta-E00) and excluding (RGB distance) the modification factors of the colors by the human perception.

The limitations of the current study were the absence of a comparison with other systems and absence of evaluation other than the effects of BBG staining of the ILM. A comparison with other systems could not be undertaken because of the limited equipment in our institute. Although structures other than the ILM should be evaluated in future studies to confirm the efficacy of image processing during HUS, the enhancement effect also could facilitate visualization of other structures, because adjustment scales are available for the color and structure in the current system to fit each aspect of the surgical procedures and the characteristics of the light source.

In conclusion, the current results strongly support the advantages of HUS vitreoretinal surgery regarding enhanced color, contrast, and brightness. Because our system differs from those described previously^1–9,16^, the same performance might not be duplicated exactly by other systems; however, any system with image-processing software is expected to improve the surgical images and help surgeons achieve better visualization.
